# Improving the Performance of an SPR Biosensor Using Long-Range Surface Plasmon of Ga-Doped Zinc Oxide

**DOI:** 10.3390/s18072098

**Published:** 2018-06-30

**Authors:** Banxian Ruan, Qi You, Jiaqi Zhu, Leiming Wu, Jun Guo, Xiaoyu Dai, Yuanjiang Xiang

**Affiliations:** 1 Engineering Technology Research Center for 2D Material Information Function Devices and Systems of Guangdong Province, College of Optoelectronic Engineering, Shenzhen University, Shenzhen 518060, China; 2161190229@email.szu.edu.cn (B.R.); 2161190234@email.szu.edu.cn (Q.Y.); 2161190233@email.szu.edu.cn (J.Z.); 1709853fii30001@student.must.edu.mo (L.W.); yjxiang@szu.edu.cn (Y.X.); 2 Laboratory of Advanced Material Photonics (LAMPs), Shenzhen University, Shenzhen 518060, China; 3 Jiangsu Key Laboratory of Advanced Laser Materials and Devices, School of Physics and Electronic Engineering, Jiangsu Normal University, Xuzhou 221116, China; guojun@szu.edu.cn

**Keywords:** SPR biosensor, long-range surface plasmon resonance, Ga-doped zinc oxide

## Abstract

Transparent conducting oxides (TCOs) have appeared in the past few years as potential plasmonic materials for the development of optical devices in the near infrared regime (NIR). However, the performance of biosensors with TCOs has been limited in sensitivity and figure of merit (FOM). To improve the performance of the biosensors with TCOs, a biosensor based on long-range surface plasmon with Ga-doped zinc oxide (GZO) is proposed. It is shown that a larger FOM with a 2~7 times enhancement compared to the traditional surface plasmon polaritons (SPPs) sensor and higher detection accuracy (DA) can be realized in our proposed sensor compared with the surface plasmon resonance (SPR) sensor with GZO. Therefore, this sensor can be used to detect biological activity or chemical reactions in the near infrared region.

## 1. Introduction

In recent years, optical sensors based on surface plasmon polaritons (SPPs) have been extensively studied. SPPs, an evanescent wave induced by the collective oscillation of free electrons on metal surfaces, is explained by the surface plasmon resonance (SPR) phenomenon using different attenuated total reflection (ATR) structures by Otto [1] and Kretschmann [2]. The surface plasmon wave (SPW), which is utilized as a probe to monitor the interaction of sensor surfaces in SPR sensors, is very sensitive to changes in the refractive index (RI). When the RI of analytes change slightly, a significant change will happen in the resonance angle. With this feature, SPR sensors are widely used in the field of environmental monitoring [3,4,5], medical sample detection [6,7], food safety [8,9,10], and biochemical applications [11,12,13].

The noble metals such as gold and silver are commonly used as SPP materials in SPR sensors. However, traditional SPR sensors based on noble metals are usually studied in the visible region. In the near infrared regime (NIR), the noble metals suffer from large optical losses, which limit the performance of plasmonic devices. Transparent conducting oxides (TCOs), which appear as candidate plasmonic materials in the NIR, have been greatly studied in recent years [14,15,16,17,18]. Compared with gold and silver in NIR, they exhibit metallic behavior but have smaller material loss [19,20]. Kim et al. [14] have demonstrated that the SPR in nanostructured TCO films in experiments and the resonance angle excited by TCO films is very broad. When it is utilized in SPR sensors, the performance of the sensor, such as its detection accuracy (DA) or FOM, is not ideal. An accurate detection of analytes and a high performance of the sensor are always pursued by researchers. Long-range surface plasmon resonance (LRSPR) is found to be an effective way to improve the performance of the sensor [21,22,23,24]. LRSPR, excited by ATR through a thin metal film wrapped between dielectrics, was first reported by Sarid [25]. In principle, the LRSPR is formed by the coupling between SPPs at both sides of the metal film only if the metal film is thin enough. Note that the dielectric constants of the two dielectrics ($\varepsilon_{1}$,$\varepsilon_{2}$) should meet the condition $\left|\varepsilon_{1}-\varepsilon_{2}\right|<<\left(\varepsilon_{1},\varepsilon_{2}\right)$ [26]. Compared with SPR sensors, the electromagnetic field intensity of the sensing surface in LRSPR sensors is enhanced by more than one order of magnitude. Hence, the LRSPR sensor is more sensitive to changes in RI of the sensing medium and, therefore, has better performance than the SPR sensor [27]. Compared to SPR sensors, the LRSPR sensor, due to its large penetration depth, can work well in detecting large-sized analytes, such as bacterial molecules with diameters of about 1 μm. In this paper, Ga-doped zinc oxide (GZO) has been chosen as the SPP material. An optical sensor based on LRSPR in NIR wavelengths has been designed.

## 2. Design Consideration and Theoretical Model

**Figure 1:**
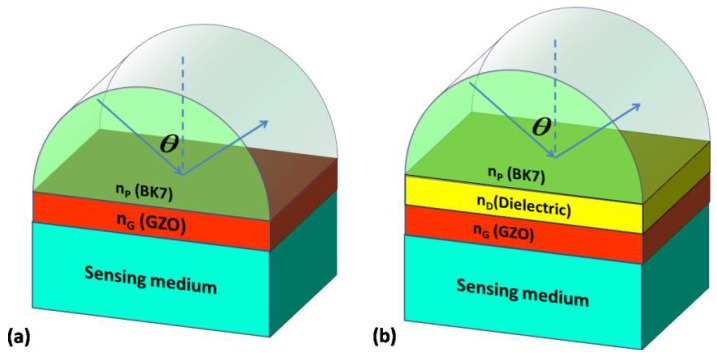
(**a**) Schematic diagram of a surface plasmon resonance (SPR) sensor based on Ga-doped zinc oxide (GZO); and (**b**) schematic diagram of a long-range surface plasmon resonance(LRSPR) sensor based on GZO.

The structures of the sensors based on SPR and LRSPR with GZO are shown in Figure 1a,b, respectively. In these structures, BK7 has been used as the coupling prism and its RI can be calculated from the following relation [28]:

(1) $$n_{Bk7}={\left(\frac{1.03961212\lambda^{2}}{\lambda^{2}-0.00600069867}+\frac{0.231792344\lambda^{2}}{\lambda^{2}-0.0200179144}+\frac{1.03961212\lambda^{2}}{\lambda^{2}-103.560653}+1\right)}^{1/2}$$

This relation shows that RI of the prism only varies with the wavelength of the incident light, and the wavelength of incident light is set to be 1550 nm. In these two types of sensors, GZO thin film has been utilized to support the excitation of SPPs and the dielectric constant can be expressed by the Drude–Lorentz oscillator model [14]:

(2) $$\varepsilon (\omega )=\varepsilon_{\infty }-\frac{\omega_{p}^{2}}{\omega (\omega +i\Gamma_{p})}+\frac{f_{1}\omega_{1}^{2}}{\omega_{1}^{2}-\omega^{2}-i\omega \Gamma_{1}}$$

where the background permittivity $\varepsilon_{\infty }=2.475,$ $\omega_{p}=1.927$ eV is the unscreened plasma frequency, $\Gamma_{p}=0.117$ eV is the carrier relaxation rate, and $f_{1}=0.866$ is the strength of the Lorentz oscillator with center frequency $\omega_{1}=4.850$ eV and damping $\Gamma_{1}=0.029$ eV.

The hybrid structure in Figure 1 with all of the layers stacking in the direction perpendicular to the prism can be solved numerically through the transfer matrix method (TMM) [29,30]. The prism and substrate are considered to be the semi-infinite layers. TMM, which uses boundary conditions of the tangential electric field and tangential magnetic field, can be utilized to analyze the reflectance R.

The change in the refractive index of the sensing medium (Δ*n*) can lead to a corresponding change in resonance angle (Δ*θ*). Hence, the figure of merit (*FOM*) is used to define the performance of the proposed sensor [21,31]:

(3) $$FOM=\frac{\Delta \theta }{\Delta n}\cdot \frac{1}{FWHM}=S\cdot DA$$

where S=Δθ/Δn is the sensitivity, DA=1/FWHM is the detection accuracy, and *FWHM* is the full width at half maximum.

## 3. Results and Discussion

**Figure 2:**
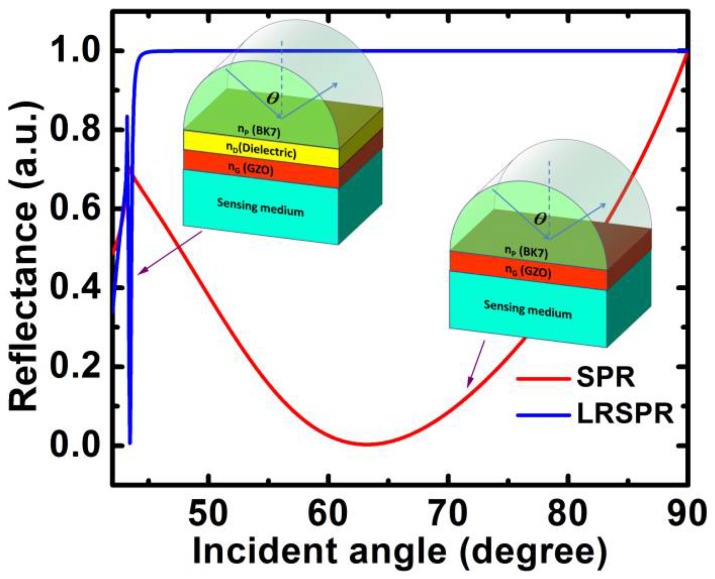
Variation in the reflectance with the incident angle for sensors based on GZO SPR and GZO LRSPR.

We can easily obtain the angular reflection spectra of the structure through TMM. In Figure 2, the red solid curve is the angular reflection spectra excited by SPPs of GZO and corresponds to the structure in Figure 1a. It can be seen that the FWHM of the resonance angle is very wide, which leads to a small FOM and DA. To overcome this disadvantage and improve the performance of the SPR sensors with GZO, we proposed a biosensor based on the LRSPR using the dielectric/GZO/sensing medium structure, as shown in Figure 1b. The blue solid curve is the angular reflection spectra with the narrower FWHM, which is excited by the LRSPR of GZO. It indicates that the FWHM can be narrowed by exciting the LRSPR with GZO; hence, a higher FOM and larger DA of the sensor can be expected.

**Figure 3:**
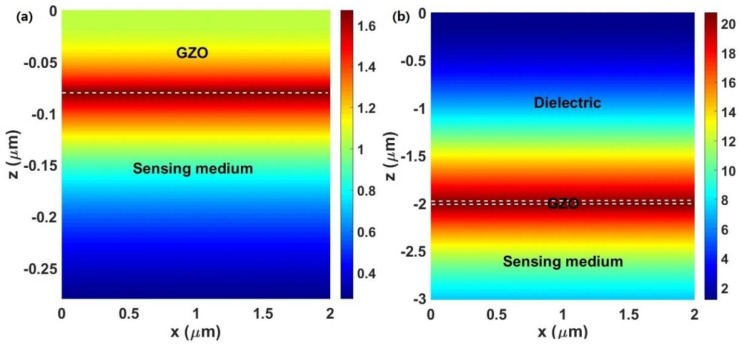
(**a**) The electric field distributions for an SPR sensor based on the 80 nm thick GZO; (**b**) the electric field distributions for a GZO-based LRSPR sensor with *d*_D_ = 2000 nm, *d*_G_ = 18 nm.

The electric field distributions for an SPR sensor and an LRSPR sensor based on GZO are shown in Figure 3. The calculated electric field is normalized by the incident electric field and z is the distance from the interface between the prism and GZO or the dielectric. From Figure 3a, a peak appears at the interface between GZO and the sensing medium while the electric field decays exponentially away from the interface, indicating the excitation of the SPP mode. Figure 3b shows the strong electric field distribution for a GZO-based LRSPR sensor with *d*_D_ = 2000 nm and *d*_G_ = 18 nm. The field can be enhanced by ~20 times in GZO-based LRSPR compared to the incident field, which is about 12 times larger than GZO-based SPR. These results suggest that the LRSPR mode is more sensitive to changes in RI in the sensing medium and, hence, can be utilized to enhance the sensitivity of a biosensor. At the same time, the penetration depth—which is defined as the distance from the interface at which the amplitude of the field decreases by a factor of 1/e [32]—can also be reflected in Figure 3. A larger penetration depth into the sensing medium of the LRSPR can be obtained [32,33,34].

**Figure 4:**
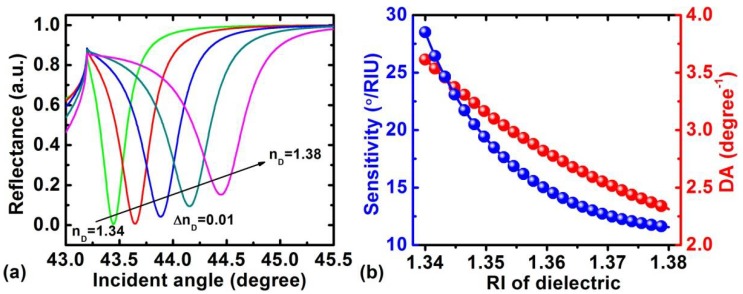
(**a**) Variation of the reflectance with the incident angle for different refractive indices(RIs) of the dielectric layer for *n_s_* = 1.33, *d_D_* = 2000 nm and *d_G_* = 18 nm; (**b**) variation in the sensitivity and detection accuracy (DA) with RI of the dielectric layer.

RI and thickness of the dielectric have a significant impact on the performance of the LRSPR sensor. To discuss the dependence of the dielectric’s RI on the performance of the sensor, the dielectric thickness was first fixed at 2000 nm. Figure 4a shows a series of ATR curves plotted as a function of $\theta_{in}$ where the RI of the dielectric varies from 1.34 to 1.38. It can be seen that with an increasing dielectric RI, the resonance angle shifts to the large incident angle and the angular reflection spectra become broader. The corresponding results are summed in Figure 4b; the larger RI of the dielectric corresponds to a smaller sensitivity and DA.

**Figure 5:**
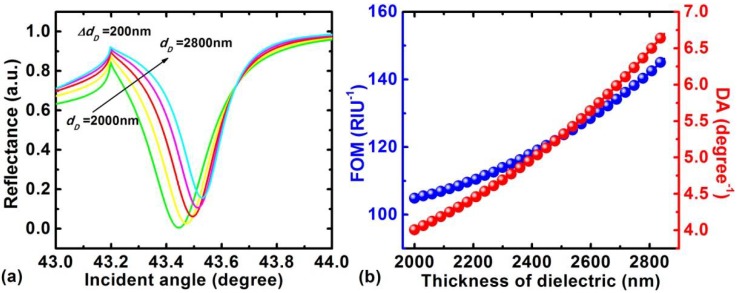
(**a**)Variation of the reflectance with the incident angle when the thickness of the dielectric (*d_D_*) changes from 2000 nm to 2800 nm; (**b**) variation of the FOM and DA with the increase of dielectric thickness.

The dependence of the LRSPR sensor on the dielectric thickness is indicated in Figure 5, where the reflectance varies with the dielectric thickness from 2000 nm to 2800 nm. It clearly shows that the resonance angle shifts to the larger incident angle and the resonance becomes narrower with the increasing dielectric thickness (Figure 5a). Furthermore, to understand the performance of an LRSPR sensor, we gave the corresponding FOM and DA in Figure 5b. It clearly shows that the larger dielectric thickness has a higher DA and FOM. Here, we found that a FOM as high as 150 RIU^−1^ can be obtained for *d*_D_ = 2800 nm.

**Figure 6:**
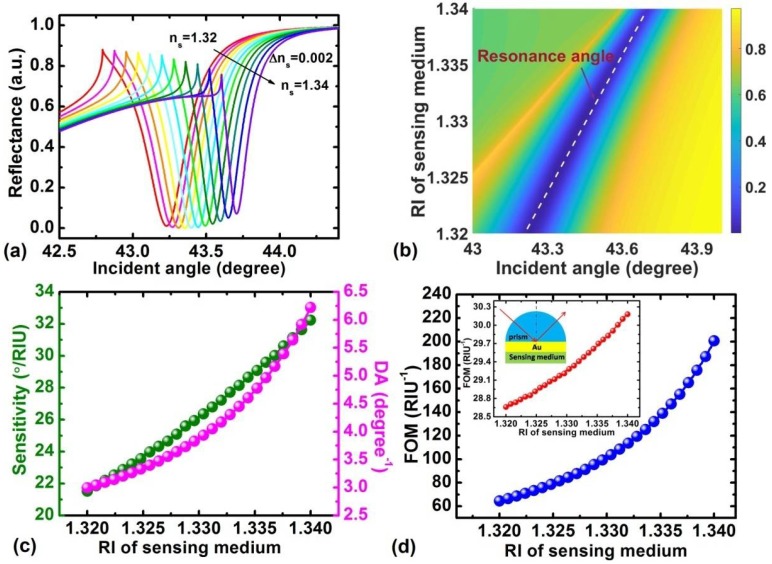
(**a**) Variation of the reflectance with the incident angle with the change of RI of sensing medium from 1.32 to 1.34; (**b**) Movement of resonance angle for the proposed GZO-based LRSPR sensor with the increase in the RI of the sensing medium; variation of sensitivity and DA (**c**), FOM of the LRSPR sensor and traditional SPR sensor (**d**) with RI of the sensing medium for a dielectric thickness of 2000 nm and GZO layer thickness of 18 nm.

The designed GZO-based LRSPR sensor is capable of detecting small changes in the refractive index. Figure 6a shows the angular reflectance spectrum for the varying RI of the sensing medium from 1.32 to 1.34. It can be seen that the resonance angle shifts to a larger angle. The movement of the resonance angle with the RI of the sensing medium and incident angle for the proposed GZO-based LRSPR sensor are shown in Figure 6b. When biological or chemical reactions occur in the sensing medium, a slight change will happen in the RI of the sensing medium. The slight change in RI in the sensing medium will have a significant effect on the performance of the proposed sensor, as demonstrated in Figure 6c,d. It shows that the variation in DA, sensitivity, and FOM with the RI of the sensing medium changes from 1.32 to 1.34 for *d_D_* = 2000 nm and *d_G_* = 18 nm. It is clear that the resonance becomes increasingly narrower with the increasing RI of the sensing medium, while DA, sensitivity, and FOM can be enhanced with the increasing RI of the sensing medium. We also compared the FOM with the traditional SPR sensors based on the 50 nm thick Au film. Compared to traditional SPR sensors, the proposed sensor achieves a significantly improved FOM, which is a 2~7 times enhanced magnitude.

## 4. Conclusions

In this paper, an optical biosensor based on LRSPR of transparent conducting oxide (GZO) has been proposed. Compared with the biosensor based on SPR of transparent conducting oxide (GZO), the performance of the LRSPR sensor is obviously improved. FOM of the proposed sensor presents an improved FOM, which is enhanced by 2~7 times compared to the traditional SPR sensor. With such improved performance, we believe that this scheme could find potential applications in chemical examination, medical diagnosis, and biological detection, etc.
